# Survival of Antibiotic Resistant Bacteria and Horizontal Gene Transfer Control Antibiotic Resistance Gene Content in Anaerobic Digesters

**DOI:** 10.3389/fmicb.2016.00263

**Published:** 2016-03-08

**Authors:** Jennifer H. Miller, John T. Novak, William R. Knocke, Amy Pruden

**Affiliations:** Virginia Tech, Charles E. Via Department of Civil and Environmental EngineeringBlacksburg, VA, USA

**Keywords:** mesophilic anaerobic digestion, thermophilic anaerobic digestion, antibiotic resistant bacteria, tetracycline, antibiotic resistance gene, horizontal gene transfer, biosolids

## Abstract

Understanding fate of antibiotic resistant bacteria (ARB) vs. their antibiotic resistance genes (ARGs) during wastewater sludge treatment is critical in order to reduce the spread of antibiotic resistance through process optimization. Here, we spiked high concentrations of tetracycline-resistant bacteria, isolated from mesophilic (Iso M1-1—a *Pseudomonas* sp.) and thermophilic (Iso T10—a *Bacillus* sp.) anaerobic digested sludge, into batch digesters and monitored their fate by plate counts and quantitative polymerase chain reaction (QPCR) of their corresponding tetracycline ARGs. In batch studies, spiked ARB plate counts returned to baseline (thermophilic) or 1-log above baseline (mesophilic) while levels of the ARG present in the spiked isolate [*tet*(G)] remained high in mesophilic batch reactors. To compare results under semi-continuous flow conditions with natural influent variation, *tet*(O), *tet*(W), and *sul1* ARGs, along with the *intI1* integrase gene, were monitored over a 9-month period in the raw feed sludge and effluent sludge of lab-scale thermophilic and mesophilic anaerobic digesters. *sul1* and *intI1* in mesophilic and thermophilic digesters correlated positively (Spearman rho = 0.457–0.829, *P* < 0.05) with the raw feed sludge. There was no correlation in *tet*(O) or *tet*(W) ratios in raw sludge and mesophilic digested sludge or thermophilic digested sludge (Spearman rho = 0.130–0.486, *P* = 0.075–0.612). However, in the thermophilic digester, the *tet*(O) and *tet*(W) ratios remained consistently low over the entire monitoring period. We conclude that the influent sludge microbial composition can influence the ARG content of a digester, apparently as a result of differential survival or death of ARBs or horizontal gene transfer of genes between raw sludge ARBs and the digester microbial community. Notably, mesophilic digestion was more susceptible to ARG intrusion than thermophilic digestion, which may be attributed to a higher rate of ARB survival and/or horizontal gene transfer between raw sludge bacteria and the digester microbial community.

## Introduction

Bacterial resistance to antibiotics is a worldwide problem resulting in untreatable infections, death, and escalating healthcare costs. A large body of research has focused on the connection between antibiotic abuse in human medicine and agricultural settings with clinical antibiotic resistance, but the spread of antibiotic resistance continues to be a problem. Thus, the landscape of the war against antibiotic resistance is expanding, with growing attention on environmental reservoirs, such as wastewater treatment plants (WWTPs). In particular, there is interest in the means by which the transfer of antibiotic resistance genes (ARGs) via environmental routes to clinical pathogens can be mitigated (Davison, 1999; Wellington et al., 2013). It is now understood that even non-pathogenic organisms, such as commensals, can be of concern and can serve as a source of ARGs to pathogenic bacteria of clinical significance (Poirel et al., 2002; Rossolini et al., 2008).

WWTPs are of interest not only because they receive wastewater laden with antibiotics, antibiotic resistant bacteria (ARBs), and the ARGs that they carry, but also because they hypothetically create a hot spot for horizontal transfer of ARGs (Dröge et al., 2000; Schlüter et al., 2007) and can elevate ARGs in impacted receiving environments (Storteboom et al., 2010; LaPara et al., 2011). The nutrient-rich, microbially-dense nature of WWTP biomass, in concert with the presence of antibiotics or other selection factors, has been linked with the elevation and persistence of ARGs in activated sludge (Stalder et al., 2013), wastewater effluent (da Silva et al., 2007; Zhang et al., 2009; Luczkiewicz et al., 2010), and land-applied biosolids (Munir and Xagoraraki, 2011; Munir et al., 2011). Early studies focused on tracking specific ARBs or ARGs in wastewater matrices, while mobile genetic elements (e.g., transposons, integrons, and plasmids) have been the subject of more recent attention given their essential role in facilitating transfer of ARGs from the environment to the clinic (Muniesa et al., 2011; Lupo et al., 2012; Moura et al., 2012; Stalder et al., 2012; Ashbolt et al., 2013; Rizzo et al., 2013; Gillings et al., 2014). The prevalence of plasmid-borne ARGs of clinical relevance in WWTPs (Szczepanowski et al., 2009) is of concern and calls for research to establish understanding of the mechanisms governing ARG persistence and proliferation and means by which to limit ARG propagation.

Sludge digestion is of particular interest as a potential means to mitigate the spread of antibiotic resistance, given that it controls the fate of the vast majority of WWTP biomass and ARGs (Munir et al., 2011). Prior studies have collectively measured tetracycline, sulfonamide, erythromycin, and Class 1 integrase genes, which are considered to be key markers of horizontal gene transfer, in mesophilic and thermophilic digestion (Ghosh et al., 2009; Diehl and LaPara, 2010; Ma et al., 2011; Miller et al., 2013). Thermophilic digestion reduced all ARGs to some extent (1–2 log), whereas mesophilic digestion had variable effects on gene levels. Ma et al. (2011) also reported an enhanced removal (2–3 log) of ARGs during thermal hydrolysis pretreatment. However, the concentration of ARGs increased, with the exception of *sul*1 and *tet*(G), in the subsequent mesophilic stage. Recent research has focused on metagenomics of ARGs and ARBs in anaerobic digestion (Resende et al., 2014; Yang et al., 2014; Zhang et al., 2015). Prior research suggests that the digester microbial community and the operating conditions play an important role in determining ARG composition and fate. However, the role of the raw sludge influent ARG composition has not previously been examined and has important implications in terms of relative importance of upstream mitigation strategies (e.g., limiting antibiotic use, separating waste streams) vs. digester operation (e.g., maintaining ideal digester microbial community by seeding or selection by temperature or other factors).

It is difficult to measure horizontal gene transfer events in complex environmental matrices (Luby et al., 2016). Rizzo et al. (2013) summarized that there are three types of retrospective evidence of horizontal gene transfer, including association of the ARG with a mobile genetic element, loss of co-location of the insertion site in the host and the acquired ARG, or the lack of similarity between the ARG phylogeny and the ARB phylogeny. Microcosm studies in sediment (Bonot and Merlin, 2010), activated sludge, biofilm, and anaerobic digestion (Merlin et al., 2011) used an increasing ratio of introduced plasmid DNA normalized to ARB chromosomal DNA over time to signify horizontal gene transfer between the introduced ARB and indigenous bacteria. In the present study, evidence of horizontal gene transfer is approached by tracking the fate of a donor ARB by plate culturing and tracking a particular ARG that it carries via quantitative polymerase chain reaction (QPCR) in experimental digesters spiked with the ARB.

The purpose of this study was to examine the effect of elevated influent ARGs originating from influent ARB hosts during mesophilic and thermophilic sludge digestion. A feature of the experimental approach was that the spiked tetracycline-resistant ARBs (designated Iso M1-1 and Iso T10) were isolated from the mesophilic and thermophilic sludge itself and thus presented the opportunity to observe the behavior of native ARBs and their ARGs when elevated in the digester environment. Iso M1-1 was isolated from mesophilic sludge and was identified as a *Pseudomonas* sp. carrying the *tet*(G) tetracycline ARG, which was monitored along with *tet*(W), an ARG not carried by the isolate and representative of the background. Iso T10 was isolated from thermophilic sludge and was identified as *Bacillus* sp. carrying *tet*(W) tetracycline ARG. Quantitative polymerase chain reaction (QPCR) of ARGs along with tetracycline-resistant plate counts provided insight into the fate of the isolate and evidence of horizontal gene transfer. To further examine the fates of ARGs under different digester operating conditions, *sul*1, *tet*(O), and *tet*(W), the *intI1* gene encoding the integrase enzyme of Class 1 integrons were monitored in influent raw sludge and digested sludge from lab-scale mesophilic and thermophilic digesters. Overall results of this study help support management options for reducing the spread of antibiotic resistance via sludge digestion.

## Materials and methods

### Batch digestion of tetracycline-resistant isolates

#### Culture media

Tryptic soy agar (TSA, 40 g/L) was added to reverse osmosis water, dissolved by heating, autoclaved at 121°C for 20 min, and cooled to 55°C in a water bath prior to antibiotic amendment. Filter-sterilized solutions of tetracycline hydrochloride (Sigma Aldrich) and cycloheximide (Sigma Aldrich) were added to the agar at final concentrations of 16 and 200 mg/L, respectively. Agar was mixed at low speed on a stir plate prior to pouring Petri dishes (100 × 15 mm). These plates were used in the isolation of tetracycline-resistant microorganisms from sludge and in monitoring their fate in batch digestion experiments.

#### Isolation of tetracycline-resistant bacteria

Tetracycline-amended (16 mg/L) TSA plates were inoculated with 100 μL of 1x, 10x, and 100x diluted mesophilic or thermophilic digested sludge and incubated at 37°C for 24 h. After 24 h, individual colonies were aseptically re-streaked on fresh tetracycline-amended TSA plates and incubated again for 24 h. This process was repeated at least 3 times for 5 mesophilic colonies and 13 thermophilic colonies. DNA extracts of all colonies were screened for *tet*(C), *tet*(G), *tet*(O), or *tet*(W) presence using QPCR because these genes have been associated with wastewater (Storteboom et al., 2010). However, *tet*(C) and *tet*(O) were not detected in any colonies. The mesophilic colony labeled Iso M1-1 was positive only for *tet*(G) and thermophilic colony labeled Iso T10 was positive only for *tet*(W). Consistent morphology and growth curves were obtained for Iso M1-1 and Iso T10 (6 and 10-h growth times to stationary phase, respectively). Freezer stocks of the isolates were prepared with cultures grown to density, concentrated by centrifugation, rinsed with phosphate buffered saline, re-concentrated, resuspended in tryptic soy broth with 20% glycerol, and stored at −80°C until use.

Cloning and sequencing of the Iso M1-1 16S rRNA gene indicated that the isolate was a *Pseudomonas* sp. (accession number KC211303.1, 99% of identity Phylum Proteobacteria, Class Gammaproteobacterium) in 20 of the 20 tested clones. The closest match to Iso T10 was *Bacillus* sp. (accession number KJ5464450.1, 99% of identity, Phylum Firmicutes) in 20 of the 20 tested clones. Cloning used 16S rRNA primers 8F and 1492R and sequencing returned ~640 basepairs spanning the V1 and partial V2 regions. These results suggest that the isolation procedure was successful in isolating only one genus from each sludge.

#### Batch digester preparation

Six hundred mL flasks of tryptic soy broth were each spiked with 1 mL of Iso M1-1 or Iso T10 freezer stock and cultured to late log phase. Cells were harvested by centrifugation of individual flask contents at 10,000 rpm for 10 min and re-suspended and rinsed in phosphate buffered saline (PBS). The rinse step was repeated three times prior to final resuspension of pellets from two flasks per biological replicate in PBS (~30 mL total per biological replicate). The absorbance at 600 nm was measured for a 10x dilution of the concentrated cell suspension. An absorbance of 1.2 approximated a cell density of 10^8^ cells per mL (10^9^ cells per mL in the undiluted suspension). Digested mesophilic or thermophilic sludge (4.5 mL) was aliquoted to sterile plastic disposable culture tubes (100 × 17 mm, Fisher Scientific) and spiked with the concentrated Iso M1-1 or Iso T10 pure culture, respectively. Additional triplicates were unamended and served as control blanks at time zero. Tubes were incubated in a water bath at the test temperature (i.e., 37or 53°C) over a range of time points up to 40 days in order to capture the death/decay curves. Triplicate sample tubes were removed at each time point and immersed in a water/ice bath to quench the temperature reaction. After quenching, plating on tetracycline-amended agar was done immediately and an aliquot of the sample was stored at −20°C for later DNA extraction of an ARG contained within the isolate and a control ARG (not contained within the isolate).

#### Cell culturing/plating

Aliquots (100 μL) of serial 10x dilutions of the original sample were plated immediately on two tetracycline-amended TSA plates and incubated at 37°C for 24 h prior to cell count. Prior to each dilution and plating, culture tubes were vortexed a moderately high speed for 30 s to disengage bacteria from sludge particles.

#### pH and TS/VS

Total and volatile solids (Method 2540-G) and pH (Method 4500) were analyzed at the start and end of the batch studies as specified in Standard Methods for the Examination of Water and Wastewater (APHA/AWWA/WEF, 1995).

### Continuous feed digester study

#### Continuous feed digester setup

One 15 L, 12-day solids retention time (SRT) thermophilic (53°C) digester and one 10 L, 20-day SRT mesophilic (37°C) digester were operated as control digesters for a series of digestion studies, as reported elsewhere (Miller et al., 2013). In brief, high density polyethylene cone fermenters (Hobby Beverage Equipment Company, Temecula, CA) with nominal volumes of 6.5 gallons (24.6 L) were fed daily with a 70% primary sludge and 30% thickened waste activated sludge mixture from the Christiansburg, Virginia WWTP, which predominantly receives residential wastewater with minimal industrial contribution and no hospital contributions. The mesophilic and thermophilic digesters were originally seeded with digested (37°C) Christiansburg WWTP sludge and laboratory-scale thermophilic (48–57°C) digester material, respectively. Feed to both digesters was diluted with tap water to maintain a consistent influent total solids (TS) of 2.5% and reduce variations in operating parameters (e.g., pH, alkalinity, VFAs, volatile solids reduction). Raw sludge was collected every 4–6 weeks and stored at 4°C to minimize biological activity until fed into the digesters. TS, volatile solids (VS), and pH of the raw sludge were routinely measured over the 9-month study period. A peristaltic pump was used to mix digester contents by recycling headspace gas to the bottom of the cone digester. Evolved gases were collected in 25-L Tedlar bags (SKC, Inc., 84, Pennsylvania). Both digesters were maintained in a 37°C constant temperature room with the thermophilic digester modified with electric heating tape with a temperature controller (Model No. BSAT 101-100, Thermolyne, Dubuque, Iowa) to further elevate the temperature. Miller et al. (2013) reports data associated with control digesters performance monitoring, including total and volatile solids, total alkalinity, pH, gas volume, headspace methane and carbon dioxide, and volatile fatty acids. DNA samples were collected then stored at −20°C for later DNA extraction from feed sludge (after refrigeration, directly prior to feeding), mesophilic control digester effluent, and thermophilic control digester effluent over the 9-month study period. ARGs [*tet*(O) and *tet*(W)] were selected for analysis because they are typically associated with wastewater sources (Storteboom et al., 2010) and they have been shown to have variable treatment efficiencies in anaerobic digestion (Diehl and LaPara, 2010; Ma et al., 2011; Miller et al., 2013). *intI1* and ARG *sul1* were selected for analysis because they are associated with Class 1 integrons (Mazel, 2006), which have been suggested as a relative indicator of antibiotic resistance (Gillings et al., 2014). In the present study, ARG data points that were used to generate previously published bar chart averages of ARG concentration (Miller et al., 2013) are combined with previously unpublished ARG data points and plotted to examine Spearman rho correlations between ARGs concentrations in the raw feed sludge and digester effluents. These correlations provide insight into the influence of the ARG content of raw sludge on the ARG content of digester effluent (biosolids).

### Statistics

Sigmaplot 11.0 (2008) was used to calculate Spearman's rho correlation coefficients and *P*-values (Helsel and Hirsch, 2002) between ARG-16S ratios measured in the raw sludge feed and digester effluent in the continuous feed digester study. If the correlation coefficient was positive, the variables tend to increase together. If the correlation coefficient was negative, the values tend to decrease together. A *P* < 0.05 was considered significant.

### Quantification of ARGs for batch and continuous feed studies

DNA was extracted from 175 μL of sludge samples using the MagMax Total Nucleic Acid Extraction Kit (Ambion, Life Technologies) according to manufacturer's protocol. Extracted DNA was diluted 50 × to minimize inhibitory effects and stored at −20°C until analysis by QPCR for bacterial 16S rRNA genes, *tet*(G), *tet*(W), *tet*(O), *sul1*, and *intI1*. A 10 μL reaction mixture was comprised of 5.0 μ L SsoFast Evagreen Supermix (Bio-Rad, Hercules, CA), 0.8 μL of each 5 μM primer (Table 1), 2.4 μL molecular biology grade water, and 1 μL of DNA template. All samples were quantified in triplicate. Standards (serial dilutions of cloned genes ranging from 10^1^ to 10^7^ gene copies per μL) and a reagent blank (reaction mixture with molecular biology grade water substituted for DNA template) were included in triplicate with each QPCR well plate. Bio-rad CFX Manager 3.0 generated a standard curve of threshold cycle against log gene concentration. Successful standard curves were characterized by *R*^2^> 0.98 and amplification efficiency between 90 and 105% and were used to calculate ARG concentration in the diluted sample extract. ARG gene copy number concentration in sludge was calculated by adjusting the diluted sample extract ARG concentration by reduced volume subsequent to DNA extraction (0.3x), dilution of the extract to reduce QPCR inhibitor concentrations (50x), and unit conversion from μL to mL (1000x). The lowest standard on the QPCR curve (10 gene copy numbers (gcn) per μL) was equivalent to 1.5 × 10^5^ gcn per mL digested sludge.

**Table 1:** **Primer sequence and annealing temperature of the QPCR assays**.

**Target Gene**	**Primer**	**Primer sequence (5′–3′)**	**Annealing temperature**	**References**
*sul*I	*sul*I–Fw	CGCACCGGAAACATCGCTGCAC	69.9	Pei et al., 2006
	*sul*I–Rv	TGAAGTTCCGCCGCAAGGCTCG	69.9	
*tet*(O)	*tet*(O)–Fw	ACGGARAGTTTATTGTATACC	50.3	Aminov et al., 2001
	*tet*(O)–Rv	TGGCGTATCTATAATGTTGAC		
*tet*(W)	*tet*(W)–Fw	GAGAGCCTGCTATATGCCAGC	60.0	Aminov et al., 2001
	*tet*(W)–Rv	GGGCGTATCCACAATGTTAAC		
*intI1*	HS463a	CTGGATTTCGATCACGGCACG	60.0	Hardwick et al., 2008
	HS464	ACATGCGTGTAAATCATCGTCG		
16S rRNA (bacterial)	1369F	CGGTGAATACGTTCYCGG	60.0	Suzuki et al., 2000
	1492R	GGWTACCTTGTTACGACTT		

## Results

### Fate of tetracycline resistant isolate (Iso M1-1) during mesophilic digestion

**Figure 1:**
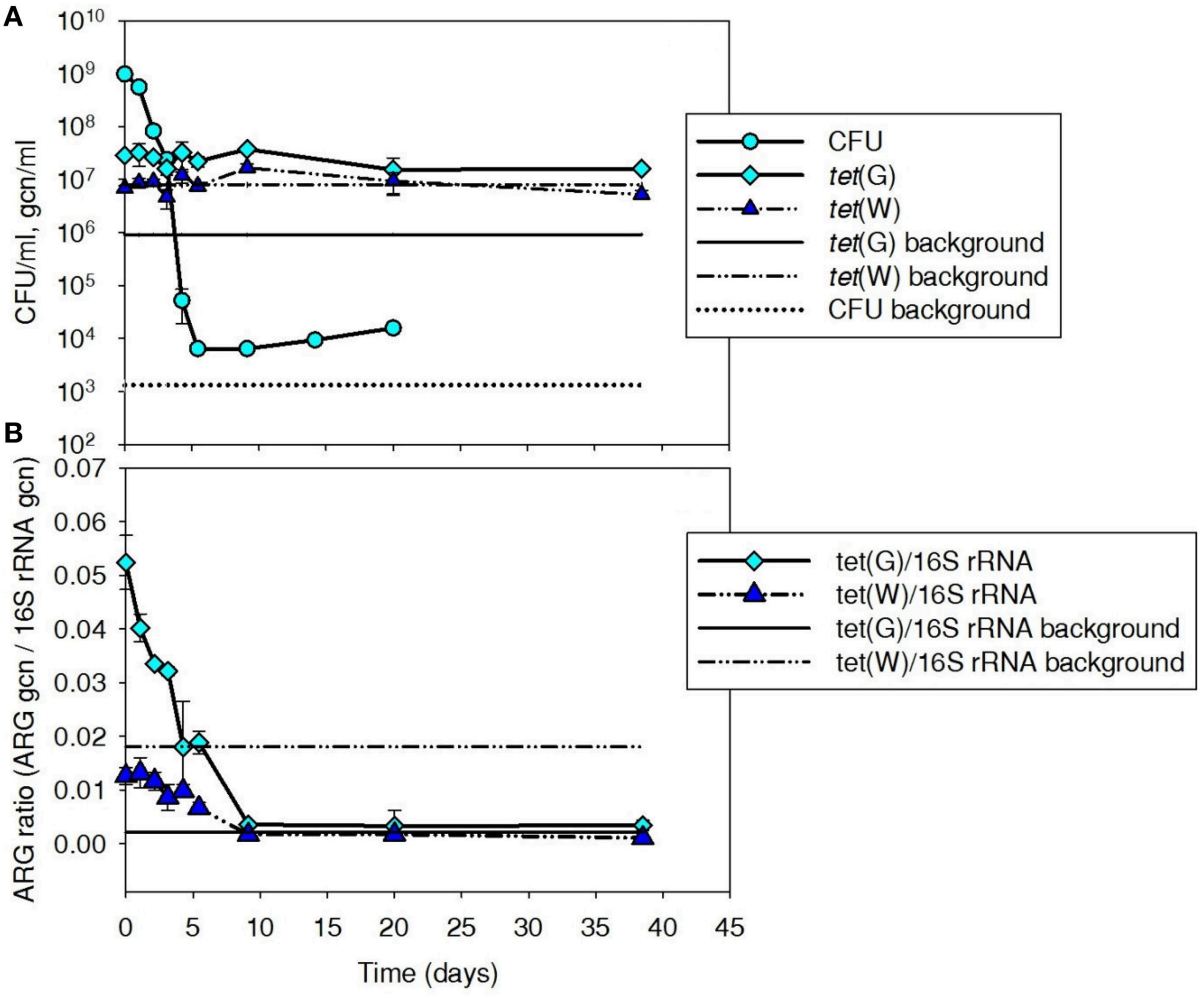
**Plate count (CFU/ml sludge) and (A) *tet*(G) and (B) normalized ARG ratios (ARG gcn/16S rRNA) for *tet*(G) associated with tetracycline resistant bacteria, including Iso M1-1, during mesophilic treatment at 37°C in digested sludge**. *tet*(W) was included as a control gene because it was not associated with Iso M1-1, but was present in the background community. Background line represents the pre-spike concentration in digested sludge.

Tetracycline-resistant CFU and *tet*(G) were monitored to provide insight into the fate of Iso M1-1 over a 40-day period after it was spiked to batch mesophilic digesters (Figures 1A,B). Because *tet*(W) was absent in Iso M1-1, it was monitored as a control ARG present in the background digester community. ARG numbers are presented both as gene copy numbers per volume of sludge (Figure 1A) and normalized to background 16S rRNA genes (Figure 1B), as an indicator of the relative proportion of bacteria carrying ARGs and a means to normalize minor variation in DNA extraction efficiency.

Tetracycline-resistant CFU were elevated by about 5-log above background (1.3 × 10^3^ CFU/ml) following the initial spike and subsequently decreased over a 5-day period to ~10^4^ CFU/ml, where they remained ~1-log above background for the remainder of the experiment. At these dilution levels, a few different cell morphologies were evident, but it was not possible to distinguish the Iso M1-1 from other background tetracycline-resistant bacteria.

Digester *tet*(G) content was elevated by about 1.5-log to 2.9 × 10^7^ gcn/ml following the initial spike of Iso M1-1. *tet*(G) normalized to 16S rRNA ratio was 0.28 in the pure culture preparation just prior to spiking, suggesting that at least 28% of the Iso M1-1 population carried *tet*(G). The percentage is likely closer to 100% [i.e., all cells carried *tet*(G)], as *Pseudomonas* spp. are reported to contain a median number of 4 copies (range is between four and seven copies) of the 16S rRNA gene per cell (Stoddard et al., 2015). The *tet*(G) content of the background digester community was 9.1 × 10^5^ gcn/ml (Figure 1A). Unlike CFUs, levels of *tet*(G) did not decrease with time after the initial spike of IsoM1-1 into the batch digesters, but remained ~1–1.5-log above the background throughout the experiment. When normalized to 16S rRNA genes, *tet*(G) ratios displayed a decreasing trend and returned to background by day 10 (Figure 1B). This is because 16S rRNA genes increased over this same period, which is suggestive of bacterial growth.

In contrast to *tet*(G), *tet*(W) was not influenced by the initial spike of Iso M1-1 and remained at or near the background level throughout the experiment (Figure 1A). This was as expected, given that IsoM1-1 does not carry *tet*(W). The 16S rRNA gene-normalized *tet*(G) and *tet*(W) ratios showed similar trends (Figure 1B).

### Fate of tetracycline-resistant isolate (Iso T10) during thermophilic digestion

**Figure 2:**
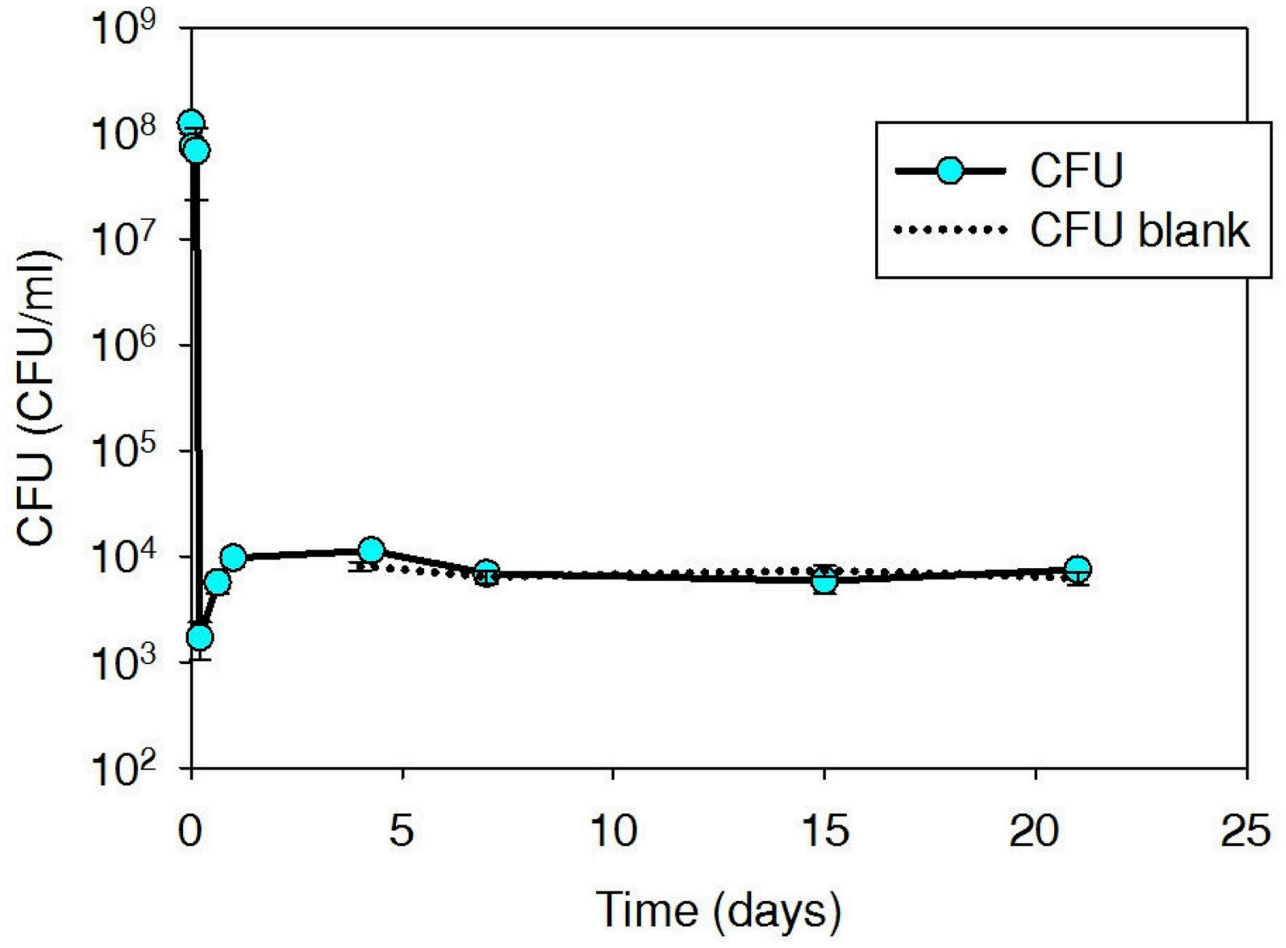
**Plate count (CFU/ml sludge) of tetracycline-resistant bacteria, including spiked Iso T10, during thermophilic treatment at 53°C in digested sludge**.

Tetracycline-resistant plate counts in initial spiked thermophilic digester microcosms were elevated to 4.0 × 10^8^ CFU/ml (Figure 2), but decreased immediately and returned to slightly above background (7.3 × 10^2^ CFU/ml) within 15 min and remained at ~10^3^ CFU/ml over an additional 40-day monitoring period. Iso T10 contained *tet*(W), not *tet*(G) (as carried by Iso M1-1). The *tet*(W) content of the background digester community was high (ranged 4.0 × 10^6^–1.2 × 10^7^ gcn/ml over the 40-day experiment), resulting in only a slight increase in *tet*(W) as a result of the spiked Iso T10, which was within the QPCR analytical error range (data not shown).

### Lab-scale semi-continuous feed digester study

**Figure 3:**
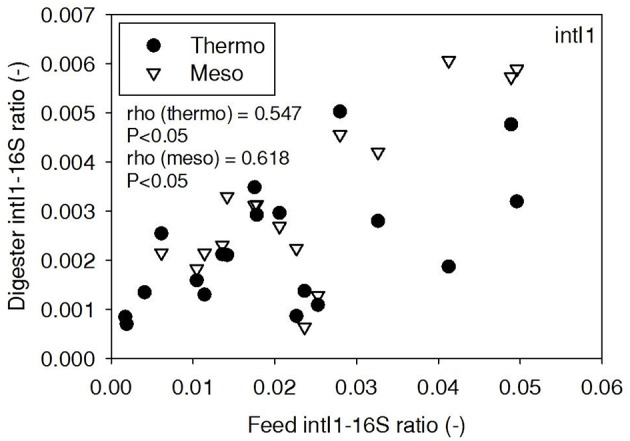
**Spearman rho correlation coefficients and *P*-values for *intI1* ARG ratios (normalized to 16S rRNA) in raw sludge feed compared to *intI1* ARG ratios in thermophilic and mesophilic digester effluent**. A subset of this data (78% of data points) were previously published in Miller et al. (2013) as bar chart ARG concentration averages in a study of the effects of antibiotic concentration on ARGs in digestion or as ARG trends with time as a function of raw sludge storage temperature.

**Figure 4:**
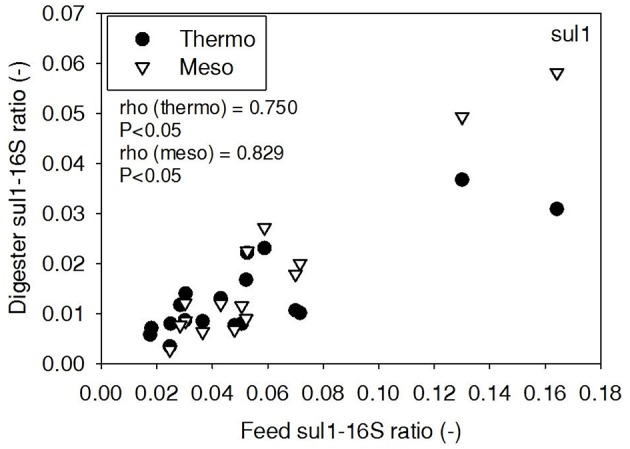
**Spearman rho correlation coefficients and *P*-values for *sul1* ARG ratios (normalized to 16S rRNA) in raw sludge feed compared to *sul1* ARG ratios in thermophilic and mesophilic digester effluent**. A subset of this data (79% of data points) were previously published in Miller et al. (2013) as bar chart ARG concentration averages in a study of the effects of antibiotic concentration on ARGs in digestion or as ARG trends with time as a function of raw sludge storage temperature.

**Figure 5:**
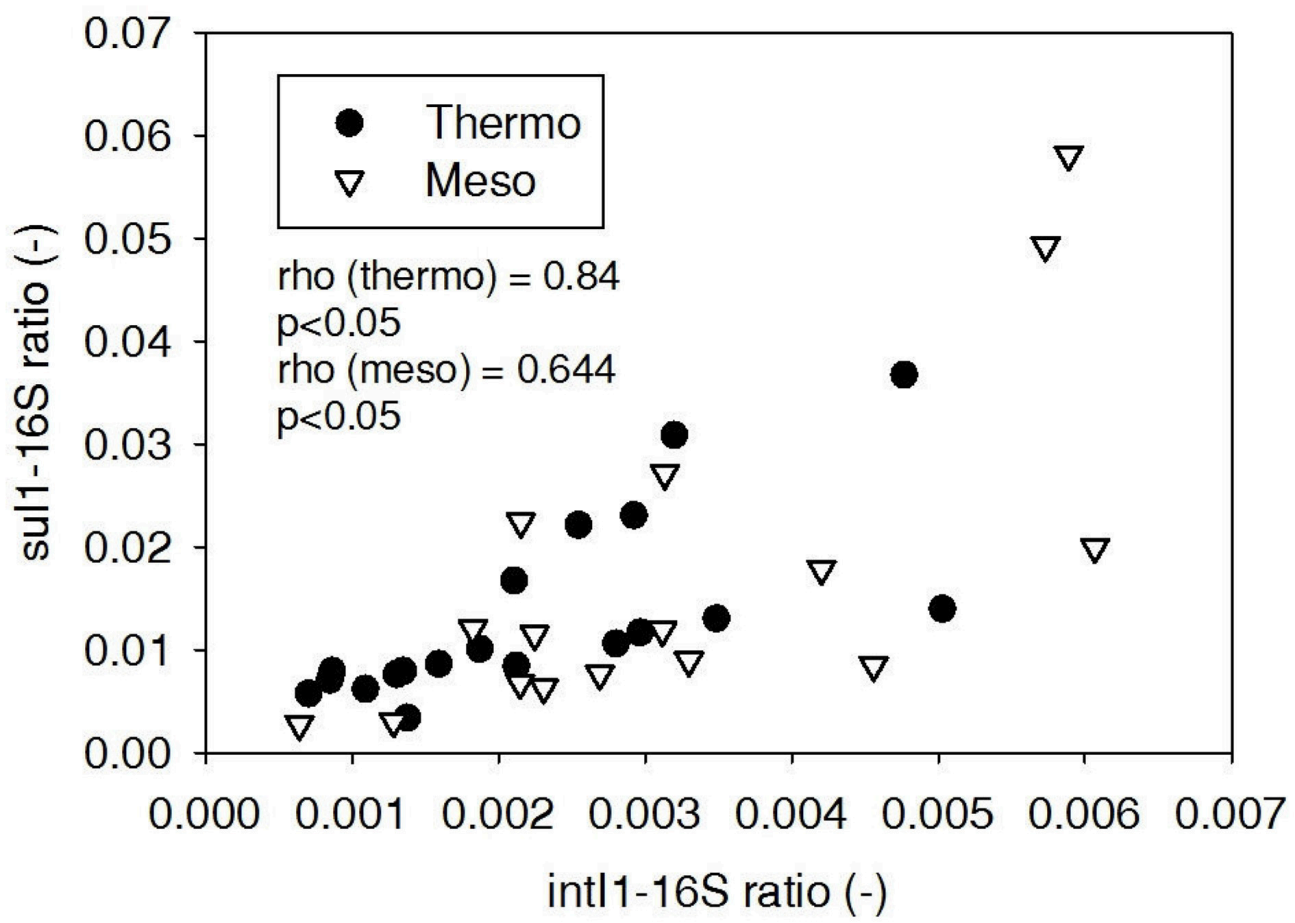
**Spearman rho correlation coefficients and *P*-values for *intI1* compared to *sul1* ARG ratios in thermophilic and mesophilic digester effluent**. A subset of this data (79% of data points) were previously published in Miller et al. (2013) as bar chart ARG concentration averages in a study of the effects of antibiotic concentration on ARGs in digestion or as ARG trends with time as a function of raw sludge storage temperature.

**Figure 6:**
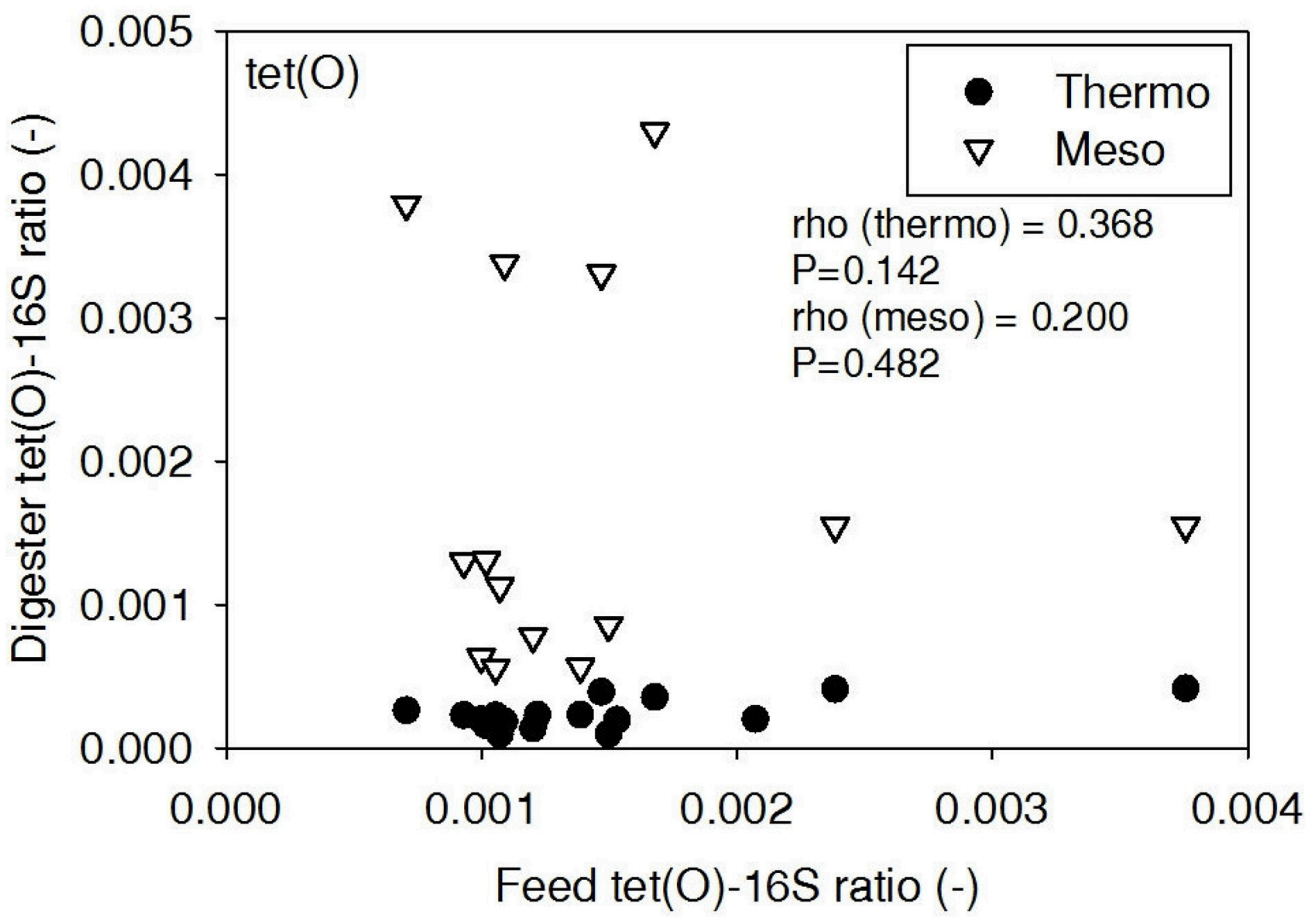
**Spearman rho correlation coefficients and *P*-values for *tet*(O) ARG ratios (normalized to 16S rRNA) in raw sludge feed compared to *tet*(O) ARG ratios in thermophilic and mesophilic digester effluent**. A subset of this data (75% of data points) were previously published in Miller et al. (2013) as bar chart ARG concentration averages in a study of the effects of antibiotic concentration on ARGs in digestion.

**Figure 7:**
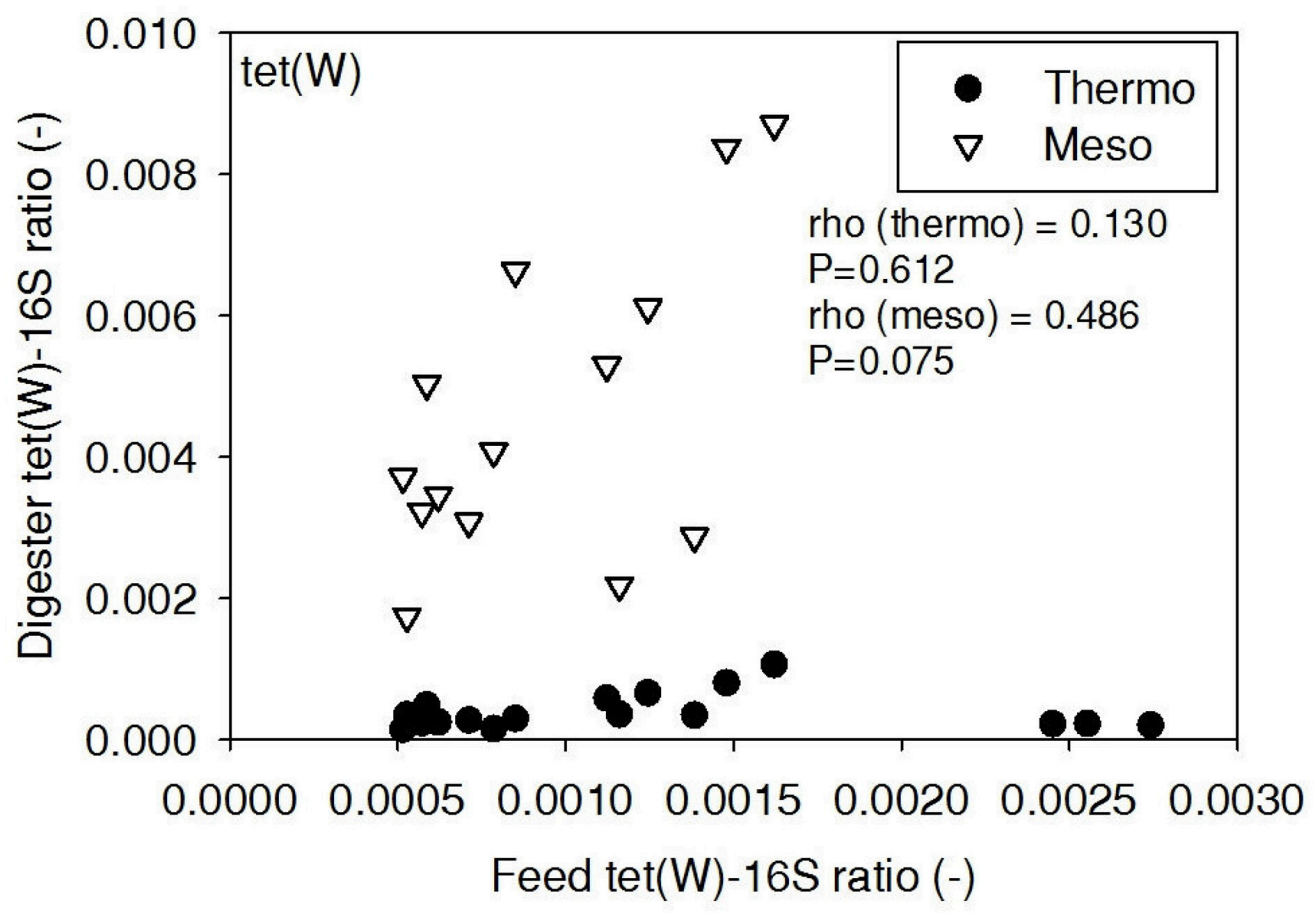
**Spearman rho correlation coefficients and *P*-values for *tet*(W) ARG ratios (normalized to 16S rRNA) in raw sludge feed compared to *tet*(W) ARG ratios in thermophilic and mesophilic digester effluent**. A subset of this data (75% of data points) were previously published in Miller et al. (2013) as bar chart ARG concentration averages in a study of the effects of antibiotic concentration on ARGs in digestion.

ARG-16S ratios in the 1) feed and ARG-16S ratios in the 2a) thermophilic digester and 2b) mesophilic digester were compared using Spearman's rho correlation analysis. *intI1* ratios in the feed sludge were positively correlated to *intI1* ratios in both thermophilic and mesophilic digesters (Figure 3). *sul1* ratios in the feed sludge were also positively correlated to *sul1* ratios in both thermophilic and mesophilic digesters (Figure 4). Interestingly, *intI1* and *sul1* ratios were positively correlated in thermophilic digesters as well as mesophilic digesters (Figure 5). This is consistent with current understanding that *sul1* is typically associated with Class 1 integrons (Mazel, 2006). In contrast, *tet*(O) and *tet*(W) ratios in the feed sludge were not correlated to *tet*(O) or *tet*(W) ratios in thermophilic or mesophilic digesters (*P* > 0.05, Figures 6, 7, respectively).

## Discussion

### Fate of tetracycline resistant isolate (Iso M1-1) during mesophilic digestion

The central question to this study is to what extent raw sludge influences the antibiotic resistance level of an anaerobic digester and its effluent? It was observed that both ARBs and ARGs survived in batch mesophilic reactors. The Iso M1-1 ARB and *tet*(G) ARG associated with this host remained 1-log and 1.5-log above background, respectively, after 40 days of digestion. This finding has important implications for the management of upstream levels of ARBs and ARGs in primary and secondary sludge, suggesting that sludge pre-treatments that aim to minimize ARB in the influent could help reduce the spread of antibiotic resistance from wastewater sources.

Tetracycline-resistant CFUs rapidly declined following spiking of Iso M1-1 in the influent, indicating that they were initially at a level greater than its carrying capacity for the digester community. This is not surprising given that digesters rely on a symbiotic balance among communities of hydrolytic, fermentatative, and methanogenic microorganisms and any one group cannot survive or thrive without the others. However, the 1-log long term increase in tetracycline-resistant CFUs also suggests that the niche filled by Iso M1-1 within the mesophilic digester community was sufficiently large to accommodate a spike in its population. However, the tetracycline-resistant plate count method could not distinguish Iso M1-1 specifically from the background resistant microorganisms. Bearing this in mind, horizontal transfer of *tet*(G) to other hosts in the digester microbial communities could have contributed to the increase in tetracycline-resistant CFU.

These results may be specific to Iso M1-1, which was native to a digested sludge environment, whereas, exogenous bacteria typically present in raw sludge may have a different fate. To our knowledge, the overlap of ARB survival or microbe composition in raw sludge and digesters has not previously been reported. However, studies examining ARG survival have reported 1- to 2-log reduction of several ARGs relative to raw sludge during thermophilic digestion, while ARG fate reported under mesophilic digestion conditions has been more variable (Ghosh et al., 2009; Diehl and LaPara, 2010; Ma et al., 2011; Miller et al., 2013). These prior studies did not track or quantify specific ARBs or evidence of horizontal gene transfer.

Sequencing of the Iso M1-1 16S rRNA gene indicated that it was a *Pseudomonas* sp. *Pseudomonas* are generally characterized as being extremely metabolically diverse with respect to capability of utilizing a wide range of organic compounds as electron donors and carbon sources and as such have been identified as key players in anaerobic digestion (Kummerer, 2009; Zheng et al., 2013). *Pseudomonas* also tend to be adept at acquiring and disseminating ARGs, with certain multi-antibiotic resistant strains particularly problematic in hospitals (Obritsch et al., 2004). They are also thought to widely carry tetracycline resistance and have been found to house two or more tetracycline ARGs at a time, including *tet*(G) (Roberts, 2015). The fact that *Pseudomonas* are typically aerobic organisms, with some using nitrate as an electron acceptor (Madigan et al., 2010), supports the conclusion that they had a relatively narrow niche in the digester community, but were able to horizontally transfer ARGs as spiked levels attenuated. Interestingly, 16S rRNA gene levels lagged in their decline following CFU decline, suggesting that some time is required for the digester community to re-appropriate cell components (DNA) from dead Iso M1-1 cells into new biomass through growth.

### Fate of *tet* ARG during mesophilic digestion

As discussed above, *tet*(G) ARGs were elevated by ~1.5 log in the effluents of the batch mesophilic digesters, presumably originating from the spiked Iso M1-1 known to be carrying it. In addition to survival of a significant fraction of IsoM1-1 cells and horizontal transfer of *tet*(G) to new hosts, persistence of extracellular *tet*(G) cannot be completely ruled out (Nielsen et al., 1996; Pietramellara et al., 2009; Dominiak et al., 2011). However, Zhang et al. (2013) quantified extracellular and intracellular DNA composition in livestock waste lagoon sludge and found that extracellular DNA accounted for less than 0.5% of the total DNA in DNA extracts from sludge, indicating that it degrades readily in sludge environments. Assuming similar distribution of DNA in digesters, *tet*(G) likely existed primarily in the intracellular form.

The maintenance of *tet*(G) at elevated levels above background combined with a substantial decrease from spiked levels in the CFU plate counts of Iso M1-1 suggest that horizontal gene transfer was likely the primary mechanism of *tet*(G) persistence. Rizzo et al. (2013) employed a similar culture-based method to assess horizontal transfer of resistance plasmids where growth of donor bacteria and transconjugates on tetracycline-amended agar plates resulted in elevated tetracycline-resistant CFUs when compared with the plates where the donors and recipients were not inoculated together. Merlin et al. (2011) observed similar loss of donor bacteria with a concurrent relative stability of plasmid pB10 DNA in batch mesophilic (35°C) anaerobic digestion experiments and concluded that the ARG plasmid had entered the indigenous background population via conjugative transfer. Interestingly, the initial 5-log increase in tetracycline-resistant CFUs (an increase of ~10^8^ CFU/ml) translated into only 2-log increase in *tet*(G) (an increase of ~10^7^ gcn/ml) relative to the background. This suggests that the shock of the initial experimental set-up may stimulate Iso M1-1 to expel a portion of the *tet*(G) that it carried.

*tet*(G) has a broad host range and is commonly associated with mobile genetic elements, which supports the conclusion that significant horizontal gene transfer contributed to its persistence in the mesophilic digesters. *tet*(G) is found in Gram negative bacteria and encodes an energy-dependent, membrane-associated efflux protein. Currently, the following genera are known to be capable of carrying *tet*(G), including *Mannheimia, Ochrobactrum, Roseobacter, Salmonella, Acinetobacter, Brevundimonas, Enterobacter, Escherichia, Fusobacterium, Pasteurella, Proteus, Providencia, Pseudomonas, Shewanella*, and *Vibrio* (Roberts, 2015). It is likely that this list is not exhaustive, but limited by the studies investigating the distribution of tetracycline resistance (Roberts, 2005). Gram negative efflux ARGs [including *tet*(G)] are commonly associated with large, typically conjugative, plasmids that often carry other ARGs (Roberts, 1996). Conjugative plasmids can be highly promiscuous and can transfer between different genera or even domains (Ochman et al., 2000). *tet* genes have not yet been found on integrons (Roberts, 2005), although *tet* genes could be located on the same transposon or plasmid as integrons (Stalder et al., 2012).

### Fate of Iso T10 and *tet* ARG during thermophilic digestion

In contrast to the mesophilic digestion condition, tetracycline-resistant CFUs returned almost immediately to background following spiking of the corresponding tetracycline-resistant ARB isolated from thermophilic sludge, Iso T10. This suggests that, in contrast to the mesophilic digestion condition, the niche for Iso T10 was not sufficient to accommodate an expanded population. This could be attributed to the fact that mesophilic digestion is characterized by a wider diversity of microorganisms that are able to assimilate, survive, and thrive in the digester environment. Likewise, it has been hypothesized that the extreme thermophilic environment limits available hosts for ARGs and may at least partially explain why thermophilic digesters often are more efficient for *tet* ARG removal (Diehl and LaPara, 2010; Ma et al., 2011; Miller et al., 2013).

Alternately, the decline in CFUs following the initial spike could be at least partially the result of bacteria entering the viable but non-culturable (VBNC) state. While it was the intent of this study to obtain isolates native to the mesophilic and thermophilic digester environments, spiking of the cultures back into the digestion tubes following several isolation steps could have affected their acclimation state to the digester environment. In particular, the thermophilic environment may have posed a shock to Iso T10, which was isolated and maintained at 37°C prior to spiking. It is also possible that Iso T10, while isolated from thermophilic digested sludge, was not a viable, active, or continuous member of the thermophilic community, but rather a transient microbe that was present during the initial plating of isolates.

### Lab-scale semi-continuous feed digester study

The lab-scale digesters afforded the opportunity to observe the influence of raw sludge ARG levels on the ARG content of mesophilic and thermophilic digesters. Over the 9-month period, feed sludge ARG levels of *sul1, intI1, tet*(O), and *tet*(W) were monitored and compared with digester effluent concentrations. Consistent with the batch-scale studies, raw sludge ARG ratios of *intI1* and *sul*1 were positively correlated with *intI1* and *sul*1 ARG ratios in both the mesophilic and thermophilic digester effluents (Figures 3, 4, respectively). This phenomenon could be explained by the survival or selective enrichment of influent raw sludge ARBs containing *intI1* or *sul*1 or by horizontal gene transfer between incoming raw sludge bacteria and the digester community. Thus, the semi-continuous feed digester study confirmed that the ARG content of raw sludge influences the ARG content of the digester effluent. Interestingly, the effect was observed for both mesophilic and thermophilic digesters.

There was no correlation of *tet*(O) and *tet*(W) ratios in raw sludge and ratios in thermophilic or mesophilic digester effluent (Figures 6, 7). Also, the *tet*(O) and *tet*(W) ratios in the thermophilic digester remained consistently low over the entire monitoring period (Figures 6, 7). These results suggest that the thermophilic digester community was relatively immune to transfer or intrusion of these *tet* ARGs or tetracycline-resistant ARBs.

In this study, raw sludge was stored in a refrigerator (4°C) to minimize biological activity (degradation of organics) prior to feeding to the digesters. Although cold storage has been shown to increase ARG concentrations in biosolids (Miller et al., 2014), the impact of cold storage on the ARB composition of the raw sludge and survivability in digestion has not been investigated. That being said, ARG concentrations reported herein were measured in raw sludge at the time of feeding (i.e., after refrigeration). Ghosh et al. (2009) monitored *tet* ARG and *intI1* in full-scale WWTPs with no cold storage of sludge prior to digestion and the *tet* ARG and *intI1* trends in thermophilic and mesophilic digestion relative to raw sludge are similar to those reported in the present study. That is, *tet* ARGs had a variable response in mesophilic digestion, whereas levels in thermophilic digestion were reduced by 0.5- to 2-log (Ghosh et al., 2009) and 1- to 2-log (Miller et al., 2013). Ghosh et al. (2009) reported that *intI1* levels were lower in mesophilic digested sludge and thermophilic digested sludge compared to raw sludge in two of three samples and three of three samples, respectively. Our work (Miller et al., 2013) has shown a consistent 1- to 1.5-log reduction of *intI1* by both mesophilic digestion and thermophilic digestion.

The variable response of different ARGs within the same digester may not be a surprise, considering *intI1* is the integrase gene associated with the highly mobile Class 1 integrons and *sul*1 is often co-located on Class 1 integrons (Paulsen et al., 1993; Zhang et al., 2009). *tet*(W) and *tet*(O) are found on plasmid and chromosomes, but have not been found within integron cassettes (Roberts, 2005). Thermophilic digestion may be able to achieve higher ARG content removal with respect to tetracycline genes because of the limited occurrence of *tet* genes within this particular thermophilic digester community, the incompatibility of the incoming raw sludge bacteria with the thermophilic digestion community for horizontal gene transfer, and/or the harsh environment restricting entrance into the digester community of new ARBs containing *tet* genes.

These results also suggest that limiting the ARG content of the initial seed community of a thermophilic digester may help limit the ARG content of digester effluent (i.e., biosolids intended for land application) because the microbial community is less prone to intrusion from raw sludge bacteria and horizontal gene transfer, thus retaining its initial ARG signature (or lack thereof). Again, these results highlight the importance of not only horizontal gene transfer, but also host survival. The limited diversity of a thermophilic digester (Wilson et al., 2008) and limiting (high temperature, high ammonia) conditions of a thermophilic digester likely limit opportunities for horizontal gene transfer and limit survival of incoming ARBs.

#### Implications for ARG management via sludge digestion

The presence of a diverse bacterial, mobile genetic element, and ARG population in activated sludge systems has recently been highlighted by metagenomic studies (Zhang et al., 2011, 2015). The activated sludge environment is characterized by ambient temperatures (10−25°C), rich nutrient and organic loading, dense microbial populations. Similar conditions also exist in mesophilic digestion, albeit with a higher and more closely controlled temperature regime (~37°C). In contrast, thermophilic regimes operate at high temperatures, endogenous conditions, with very high ammonia concentrations. Thermophilic digestion environments have been shown to support a less diverse microbial population than mesophilic digestion (Wilson et al., 2008). Moreover, to gain critical time-temperature requirements for production of Class A biosolids classification, thermophilic digesters are operated with minimum retention times prior to effluent withdrawal and shorter SRTs. This may allow longer times of uninterrupted degradation between raw sludge additions and shorter solids retention times for HGT to occur. These environmental and operating conditions may give thermophilic digestion a competitive advantage over activated sludge or mesophilic digestion in the reduction of ARG content of biosolids. Although, Zhang et al. (2015) reported similar total tetracycline ARG abundance in thermophilic and mesophilic digested sludge in a metagenomic analysis that gave a broad picture of ARG behavior, this statement is supported by our work, along with other QPCR-based studies reporting enhanced reductions of ARGs by thermophilic digestion relative to mesophilic digestion (Ghosh et al., 2009; Diehl and LaPara, 2010; Ma et al., 2011; Miller et al., 2013). Moreover, although horizontal gene transfer continues to be an important mechanism of ARG persistence for genes associated with Class 1 integrons (*sul*1, *intI1*), we also report that thermophilic digestion may allow for the reduction of other ARGs [(*tet*(O), *tet*(W)] through incoming ARB death and narrowed host range for horizontal gene transfer.

One possibility would be to intentionally seed a digester with ARG-free microorganisms to support an ARG-free effluent (biosolid). However, it is evident that horizontal gene transfer still does occur, particularly with integrons that are typically associated with mobile genetic elements, so that complete reduction of all ARGs may not be possible with ARG-free digester seed alone. Further investigation into pre-treatments designed for complete cell lysis and DNA degradation in raw sludge are warranted for both digestion methods to reduce intrusive ARBs and transformable DNA. It seems plausible that due to the apparent limitations on microbial diversity and the resultant limitations on horizontal gene transfer, that thermophilic digestion could achieve a higher rate of ARG reductions than mesophilic digestion particularly in the face of inefficient pre-treatments. In conclusion, influent ARB and ARG composition and sludge digestion conditions are important for determining the fate of influent ARBs and ARGs and ultimately determining digester effluent ARG content.

## Author contributions

JM conducted the experiments, wrote the original manuscript, and edited the manuscript. AP provided technical oversight and critical manuscript review and editing. All authors discussed the results and implications and commented on the manuscript at all stages.

## Funding

This work was supported by National Science Foundation Chemical, Bioengineering, and Transport Systems CAREER award #0852942 and Virginia Tech Institute for Critical Technology and Applied Science seed funding and award TSTS 11–26. JM was supported by the Charles E. Via, Jr. Department of Civil and Environmental Engineering Via Scholarship, Virginia Tech Graduate School Cunningham Fellowship, and Water Environment Research Foundation (WERF) Project U1R12. The findings of this study do not necessarily reflect the views of the supporting entities.

### Conflict of interest statement

The authors declare that the research was conducted in the absence of any commercial or financial relationships that could be construed as a potential conflict of interest.

## Abbreviations

ARG: antibiotic resistance gene
DNA: deoxyribonucleic acid
MRSA: methicillin-resistant *Staphylococcus aureus*
PBS: phosphate buffered saline
QPCR: quantitative polymerase chain reaction
VBNC: viable but non-culturable
USEPA: United States Environmental Protection Agency.
